# The Role of Esketamine in Reducing Propofol-Related Adverse Events During Gastrointestinal Endoscopy: A Systematic Review and Meta-Analysis

**DOI:** 10.7759/cureus.99619

**Published:** 2025-12-19

**Authors:** Lin Ba, Na Xu, XiaoXiao Dong

**Affiliations:** 1 Anesthesiology, Binzhou People's Hospital, Binzhou, CHN; 2 Endoscopy Center, Binzhou People's Hospital, Binzhou, CHN; 3 Gastroenterology, Binzhou People's Hospital, Binzhou, CHN

**Keywords:** adverse events, esketamine, gastrointestinal endoscopy, meta-analysis, propofol, sedation

## Abstract

Propofol is commonly used for sedation in gastrointestinal endoscopy, but its use is associated with dose-dependent adverse effects. Esketamine, an N-methyl-D-aspartate (NMDA) receptor antagonist, may mitigate these risks when used as an adjunct. We conducted a systematic review and meta-analysis of randomized controlled trials (RCTs) comparing esketamine plus propofol versus propofol alone in adults undergoing gastrointestinal endoscopy. We systematically searched PubMed, Web of Science, EMBASE, and the Cochrane Library up to November 29, 2025. The primary outcome was the incidence of propofol-related cardiorespiratory adverse events (hypotension, respiratory depression, bradycardia). Data were pooled using a random-effects model. Risk of bias was assessed with the Cochrane risk-of-bias tool for randomized trials (RoB 2).

Six RCTs (n = 1,199 patients) were included for qualitative synthesis. Three RCTs (n = 524) provided data for meta-analysis. The pooled analysis demonstrated that esketamine significantly reduced the risk of propofol-related adverse events (relative risk (RR) = 0.43, 95% confidence interval (CI): 0.23-0.82). Heterogeneity was high (I² > 50%). Most studies had a low risk of bias, and sensitivity analysis confirmed the robustness of the findings. Esketamine appears to mitigate propofol-associated adverse events, including hypotension, bradycardia, and respiratory depression, during gastrointestinal endoscopy. Nevertheless, further well-designed, large-scale RCTs are needed to validate these findings and determine optimal dosing strategies.

## Introduction and background

Gastrointestinal endoscopy, including procedures such as esophagogastroduodenoscopy (EGD), is a commonly performed diagnostic and therapeutic intervention. Propofol, with its rapid onset and short duration of action, has become a widely used agent for achieving the deep sedation often required to ensure patient comfort, safety, and procedural success. However, even at typical sedation doses (e.g., 1.5-2.5 mg/kg for induction), it is associated with dose-dependent adverse effects, most notably hypotension and respiratory depression [1].

While many patients tolerate these procedures well with moderate sedation (e.g., using opioids or benzodiazepines), the introduction of the endoscope beyond the upper esophageal sphincter and through the pylorus can be particularly challenging. For certain populations - such as hemodynamically unstable individuals, those at high risk for obstruction or aspiration, extremely anxious patients, or pediatric cases - deep sedation or general anesthesia is often necessary [2]. Bradycardia, while less common during brief procedural sedation, becomes a greater concern with prolonged infusion or high total doses, particularly in the context of propofol infusion syndrome. These events can lead to serious complications, particularly in elderly patients or those with comorbidities [3].

Various adjunctive agents, including opioids and midazolam, have been employed to decrease propofol requirements; however, their use is associated with adverse effects such as respiratory depression and prolonged recovery  [4]. As the S-enantiomer of ketamine, esketamine functions by antagonizing N-methyl-D-aspartate (NMDA) receptors, producing potent analgesic and sedative effects. Pharmacologically, esketamine exhibits approximately twice the affinity for the NMDA receptor compared to racemic ketamine, leading to more potent analgesia and possibly a reduced incidence of certain psychomimetic side effects (e.g., vivid dreams, agitation) at equianalgesic doses. Its primary advantages in procedural sedation include effective analgesia, preservation of respiratory drive, and stabilization of hemodynamics (e.g., mitigating propofol-induced hypotension), making it a potentially ideal adjunct to propofol.

However, esketamine is not devoid of adverse effects. Common side effects include transient hypertension, tachycardia, nausea, vomiting, dizziness, and dissociative symptoms (e.g., feeling detached from reality), which are typically dose-dependent and short-lived. Compared to racemic ketamine, esketamine may offer a marginally improved therapeutic index with faster onset and recovery, but direct comparative data in endoscopic sedation remain limited. Contraindications to its use include uncontrolled hypertension, significant cardiovascular disease (e.g., angina, heart failure), elevated intracranial or intraocular pressure, and a history of psychotic disorders. Caution is also advised in patients with hepatic impairment or a history of substance abuse [5].

Recent randomized controlled trials (RCTs) have explored the combination of esketamine and propofol in gastrointestinal endoscopic procedures, but their results have been inconsistent. Some studies report significant reductions in adverse events [6-8], while others show no clear benefit. Therefore, a systematic synthesis of the current evidence is needed to clarify the role of esketamine in this context. Hence, this systematic review and meta-analysis aimed to evaluate whether the addition of esketamine to propofol-based sedation reduces propofol-related adverse events during gastrointestinal endoscopy compared with propofol alone.

## Review

Materials and methods

This review was conducted according to the PICO framework - Population (P): Adult patients (≥18 years) undergoing sedation for elective gastrointestinal endoscopy. Intervention (I): Sedation using propofol in combination with esketamine. Comparator (C): Sedation using propofol alone (with or without a placebo). Outcomes (O): The primary outcome was the incidence of a composite of propofol-related cardiorespiratory adverse events (hypotension, respiratory depression/hypoxemia, bradycardia). Secondary outcomes included sedation quality, recovery time, patient and clinician satisfaction, and total propofol consumption.

Eligibility Criteria

Inclusion criteria: RCTs that compared I versus C in P and reported on O were deemed eligible. A minimum follow-up period of 24 hours was required to capture delayed adverse events.

Exclusion criteria: Studies were excluded if they involved non-adult populations, utilized non-RCT designs (e.g., cohort studies, case reports), did not report any of the relevant outcomes, or were not available as full-text articles.

The primary outcome for this meta-analysis was a composite of propofol-related cardiorespiratory adverse events, including hypotension, respiratory depression (or hypoxemia), and bradycardia. The specific definitions for these events (e.g., systolic blood pressure < 90 mmHg or a > 20% decrease from baseline; SpO_2_ < 90% or < 95%; heart rate < 50 beats per minute) were adopted as reported in each study.

A systematic literature search was conducted on November 29, 2025, across four electronic databases: PubMed, Web of Science, EMBASE, and the Cochrane Library. The search strategy was designed based on the PICO framework - Population/Intervention: Terms related to gastrointestinal endoscopy (endoscop*, gastroscop*, colonoscop*) AND sedation (sedation, anesthesia). Intervention/Comparator: Terms for the drug regimens: (esketamine OR S-ketamine) AND propofol.

Study Design

To restrict results to RCTs, study design-related terms (randomized controlled trial, RCT, randomly) were applied. Within each concept, terms were combined using the Boolean operator “OR,” and the main PICO components were combined using “AND.” Two reviewers independently screened titles and abstracts. Discrepancies were resolved through consensus. Of the six initially included RCTs, three were subsequently excluded from the quantitative synthesis (meta-analysis) as they were identified as study protocols without reported outcome data suitable for pooling. Thus, three studies providing complete outcome data were included in the final meta-analysis.

The Cochrane risk-of-bias tool for randomized trials (RoB 2) was used to assess the methodological quality of all six included studies. Data on study characteristics, participant demographics, interventions, and outcomes were extracted using a standardized form. Meta-analysis was performed using R software. Heterogeneity was assessed using I². A random-effects model was applied due to anticipated clinical heterogeneity. Publication bias was planned to be assessed using funnel plots; however, due to the small number of included studies (n < 10), statistical tests for funnel plot asymmetry (such as Egger's test) were not performed. Sensitivity analysis was conducted using the leave-one-out method. The decision to perform the meta-analysis with three studies is methodologically acceptable when the studies are homogeneous in their PICO elements and aim to address a specific clinical question, providing a preliminary quantitative summary [9]. For studies with multiple esketamine dose groups, the data from all experimental groups were combined into a single 'esketamine' group to create a single pairwise comparison against the control group, as recommended in the Cochrane Handbook.

Results

**Figure 1:**
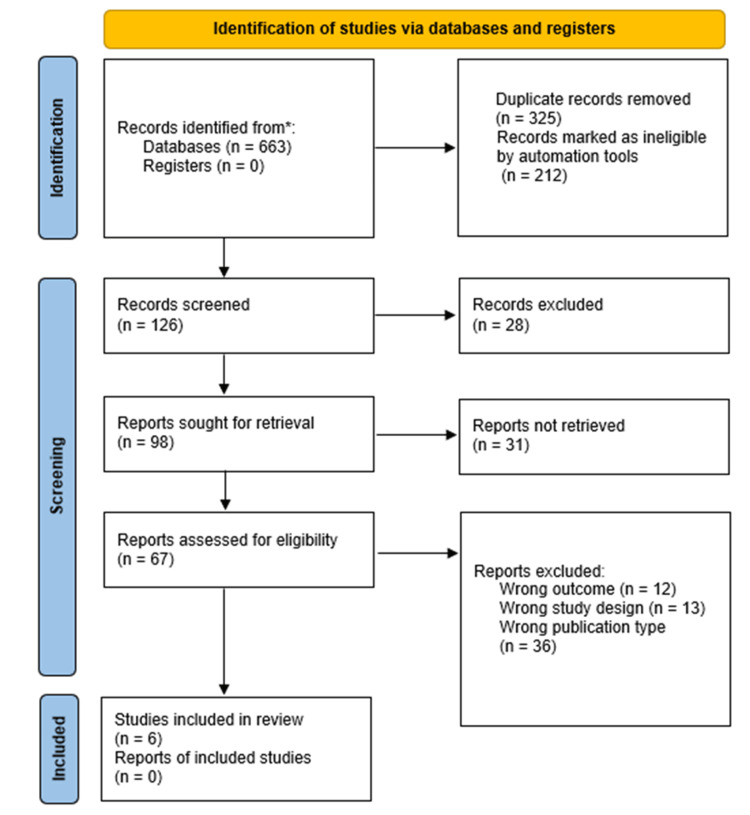
PRISMA flow diagram depicting the study selection process *A systematic search was conducted in PubMed, Web of Science, EMBASE, and the Cochrane Library This figure illustrates the flow of studies through the systematic review process, including identification, screening, eligibility, and inclusion stages PRISMA: Preferred Reporting Items for Systematic Reviews and Meta-Analyses

The study selection process is detailed in the PRISMA (Preferred Reporting Items for Systematic Reviews and Meta-Analyses) flow diagram (Figure 1). The systematic search identified six RCTs published between 2022 and 2025 for qualitative synthesis. A total of 1,199 participants were enrolled across these studies. The characteristics of all six studies are summarized in Table 1. For the quantitative synthesis (meta-analysis), three studies [7-8,10] were included, encompassing 524 patients (Zhan et al., n = 260; Zheng et al., n = 104; Ma et al., n = 160). The remaining three studies were excluded from the pooled analysis for the following reasons: two studies were study protocols without available results [6,11], and one study employed a three-arm design (comparing esketamine+propofol, sufentanil+propofol, and propofol alone), which did not align with the direct two-arm comparison (esketamine+propofol vs. propofol alone) specified in our protocol [12].

**Table 1:** Characteristics of the included studies for the meta-analysis Data are presented as reported in the original studies. Continuous variables are shown as mean ± standard deviation or range, as available. The outcome descriptions have been summarized for brevity. Please refer to the original publications for complete details on outcome measures and definitions (e.g., specific thresholds for hypotension and hypoxemia). This table summarizes the key features of all six randomized controlled trials identified in the systematic review. The three studies included in the quantitative synthesis are Ma et al. [10], Zheng et al. [8], and Zhan et al. [7]. The three studies excluded from the meta-analysis are Long et al. [6] and Li et al. [11], and Xiao et al. [12]. Group notations (e.g., P, PK, CS, DS) are as defined in the original studies ASA: American Society of Anesthesiologists (Physical Status Classification System); SBP/DBP/MAP: systolic/diastolic/mean arterial blood pressure; HR: heart rate; SpO₂: peripheral capillary oxygen saturation; PONV: postoperative nausea and vomiting; MMSE: Mini-Mental State Examination; OSA: obstructive sleep apnea; STOP-Bang: a screening tool for OSA (snoring, tiredness, observed apnea, pressure - high blood pressure, body mass index, age, neck circumference, gender); ciprofol: a novel intravenous sedative-hypnotic agent

Study	Region	Number of participants	Sex	Age	Population	Intervention exposure	Comparator context	Outcome	Study design
Long et al., 2022 [6]	China	180			Adult patients scheduled for same-day bidirectional endoscopy under sedation	Esketamine combined with ciprofol or propofol for sedation	Normal saline placebo combined with ciprofol or propofol	The study's primary endpoint was the incidence of a composite of hypoxemia (SpO₂ < 95%) and hypotension (MAP < 65 mmHg or a ≥20% reduction from baseline). A comprehensive set of secondary measures was also evaluated, addressing perioperative safety (e.g., significant hemodynamic and respiratory events), patient comfort (e.g., adverse effects, pain, and fatigue), and the satisfaction of both the operator and the patient	RCT
Ma et al., 2024 [10]	China	160	Male: 97, female: 63	60.5 ± 10.8 years (fentanyl group), 61.0 ± 11.1 years (esketamine group)	Patients classified as ASA physical status I-III undergoing curative endoscopic resection for colorectal lesions	Group A (fentanyl group): propofol + fentanyl; Group E (esketamine group): propofol + esketamine	Primary outcome: total propofol consumption (significantly lower in Group E). Secondary outcomes: incidence of hypotension and bradycardia (significantly lower in Group E); induction time, recovery time, patient and endoscopist satisfaction (no significant differences); other adverse events (e.g., oxygen desaturation, PONV) (no significant differences)	Group E (n = 81) demonstrated a significant reduction in both total propofol consumption (300 mg vs. 350 mg) and the incidence of hypotension and bradycardia compared to Group A (n = 79) among the 160 participants. All other outcomes, including additional adverse events, procedural timings, and satisfaction measures, were comparable between the two groups	RCT
Zheng et al., 2023 [8]	China	104	34 males and 18 females in Group C; 32 males and 20 females in Group S	Mean age: 41.1 ± 7.9 years in Group C and 42.2 ± 9.3 years in Group S	Patients with obesity undergoing painless gastroscopy	Group S: propofol + esketamine; Group C: propofol + placebo	Propofol consumption, Induction time, postoperative awakening time, orientation recovery time, hemodynamic parameters (SBP, DBP, HR, SpO_2_), incidence of adverse events (injection pain, hypoxemia, hypotension, bradycardia, choking, body movement), satisfaction scores of endoscopist and anesthesiologist	Compared to Group C, Group S required less propofol (201.3 ± 16.6 mg vs. 274.4 ± 22.6 mg), achieved quicker induction and awakening (17.8 ± 1.9 s vs. 25.4 ± 2.3 s and 4.8 ± 1.3 min vs. 6.2 ± 1.1 min, respectively), and showed more stable hemodynamics. The rate of various adverse events was significantly lower in Group S, leading to enhanced satisfaction among both endoscopists (4.58 ± 0.49 vs. 3.71 ± 0.83) and anesthesiologists (4.75 ± 0.44 vs. 3.33 ± 0.92)	RCT
Zhan et al., 2022 [7]	China	260	Male: 122, female: 138	Range: 18-60 years, mean: approximately 44 years	Patients undergoing painless gastrointestinal endoscopy	PK1 (propofol + esketamine 0.05 mg/kg), PK2 (propofol + esketamine 0.1 mg/kg), PK3 (propofol + esketamine 0.2 mg/kg)	Propofol consumption per minute, hemodynamic index, induction time, procedure time, orientation recovery time, awakening status, adverse events, Mini-Mental State Examination (MMSE) results	Compared to Group P (11.78 mg/min), propofol consumption was significantly lower in Group PK2 (10.14 mg/min; -13.92%, p = 0.021) and Group PK3 (9.57 mg/min; -18.76%, p = 0.000). While induction caused a significant drop (p = 0.000) in SBP, DBP, and HR across all groups, no significant differences were found between the groups for these parameters or for stable SpO₂ levels	RCT
Xiao et al., 2024 [12]	China	195	30 males and 35 females in Group DS, 29 males and 36 females in Group CS1, 25 males and 40 females in Group CS2	Participants aged 18-60 years	Patients undergoing painless colonoscopy	Group CS2: esketamine + propofol; Group DS: propofol; Group CS1: sufentanil + propofol	Incidence of hypoxemia, incidence of hypotension, incidence of bradycardia, incidence of hypertension, excellent and good rates of anaesthesia, perioperative changes in vital signs (MAP, HR, SpO_2_), anesthesia induction time, dischargeable time, patient satisfaction scores, endoscopist satisfaction scores, incidence of postoperative nausea and vomiting (PONV), incidence of drowsiness, incidence of dizziness, incidence of propofol injection pain, incidence of assisted ventilation, incidence of vasoactive medication usage (ephedrine, nitroglycerin, atropine)	Key outcomes distinctly favored the CS2 group. The DS group presented with the highest overall burden of adverse events, showing significantly greater rates of intraoperative hypoxemia, hypotension, propofol injection pain, assisted ventilation, ephedrine use, and drowsiness compared to the other groups. The risk of hypoxemia and hypotension was 5.7 and 9.9 times higher in DS than in CS2, respectively. While the CS1 group had a lower risk profile than DS, its risk of hypotension was still 5.2 times that of CS2, and it reported a significantly higher incidence of dizziness during recovery	RCT
Li et al., 2025 [11]	China	294		Participants aged 18-90 years	Patients with moderate-to-high risk OSA, defined by a STOP-Bang score of ≥3	Low-dose esketamine + propofol; saline placebo + propofol	The primary outcome is the incidence of hypoxemia (SpO_2_ < 90% for > 10 seconds). Secondary outcomes include severe hypoxemia, duration of hypoxemia, emergency airway management, propofol consumption, hemodynamic stability, involuntary body movements, procedure/recovery times, and clinician satisfaction (measured via 10-cm Visual Analog Scale)		RCT

**Figure 2:**
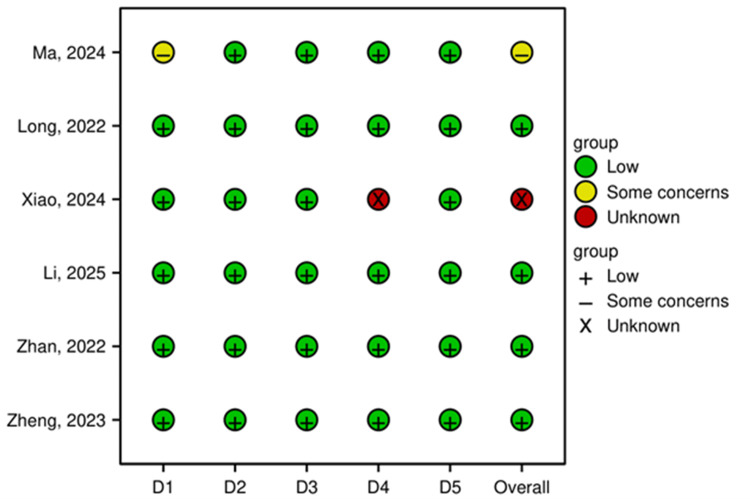
Risk of bias summary This figure summarizes the risk of bias judgments for each domain of the Cochrane RoB 2 tool across all six included studies [6-8,10-12]. The domains are as follows: D1: randomization process; D2: deviations from intended interventions; D3: missing outcome data; D4: measurement of the outcome; and D5: selection of the reported result

**Figure 3:**
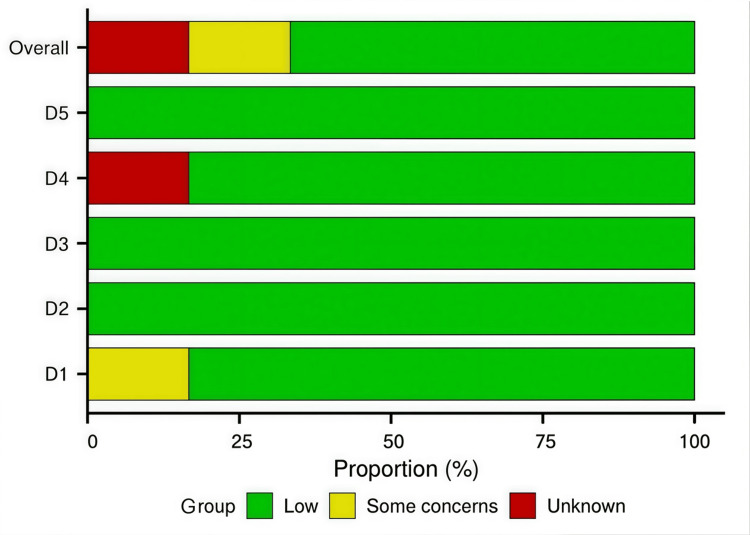
Risk of bias results This graph presents the proportion of studies with low risk, some concerns, or high risk of bias for each domain across all six included studies

A total of six studies were assessed for risk of bias using the RoB 2 tool. The overall risk of bias was low in four studies (66.7%), while one study (16.7%) raised "some concerns," and one (16.7%) had an "unknown" risk due to insufficient reporting. The domain-specific assessment is summarized in Figure 2. Figure 3 presents the results related to risk of bias.

**Figure 4:**
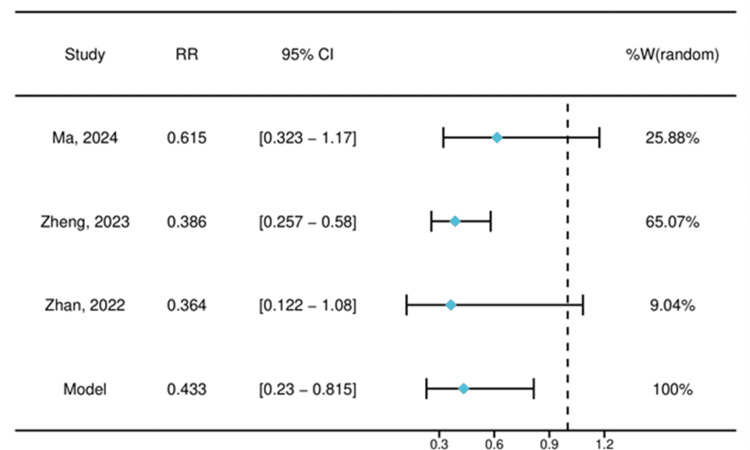
Forest plot of the pooled effect size (risk ratio) for adverse events The forest plot displays the RR and 95% CI for each of the three included studies and the pooled result. Ma et al. (2024) [10]: RR: 0.62, 95% CI: 0.32-1.17; Zheng et al. (2023) [8]: RR: 0.39, 95% CI: 0.26-0.58; Zhan et al. (2022) [7]: RR: 0.36, 95% CI: 0.12-1.08. The diamond represents the pooled estimate (RR: 0.43, 95% CI: 0.23-0.82) RR: risk ratio; CI: confidence interval

The forest plot (Figure 4) summarizes the meta-analysis of three studies [7-8,10] evaluating the effect of esketamine on propofol-related adverse events. The pooled relative risk, as estimated by a random-effects model, was 0.433 (95% CI: 0.230-0.815), indicating a statistically significant reduction in adverse events associated with esketamine. The heterogeneity among the studies was high (I² > 50%).

**Figure 5:**
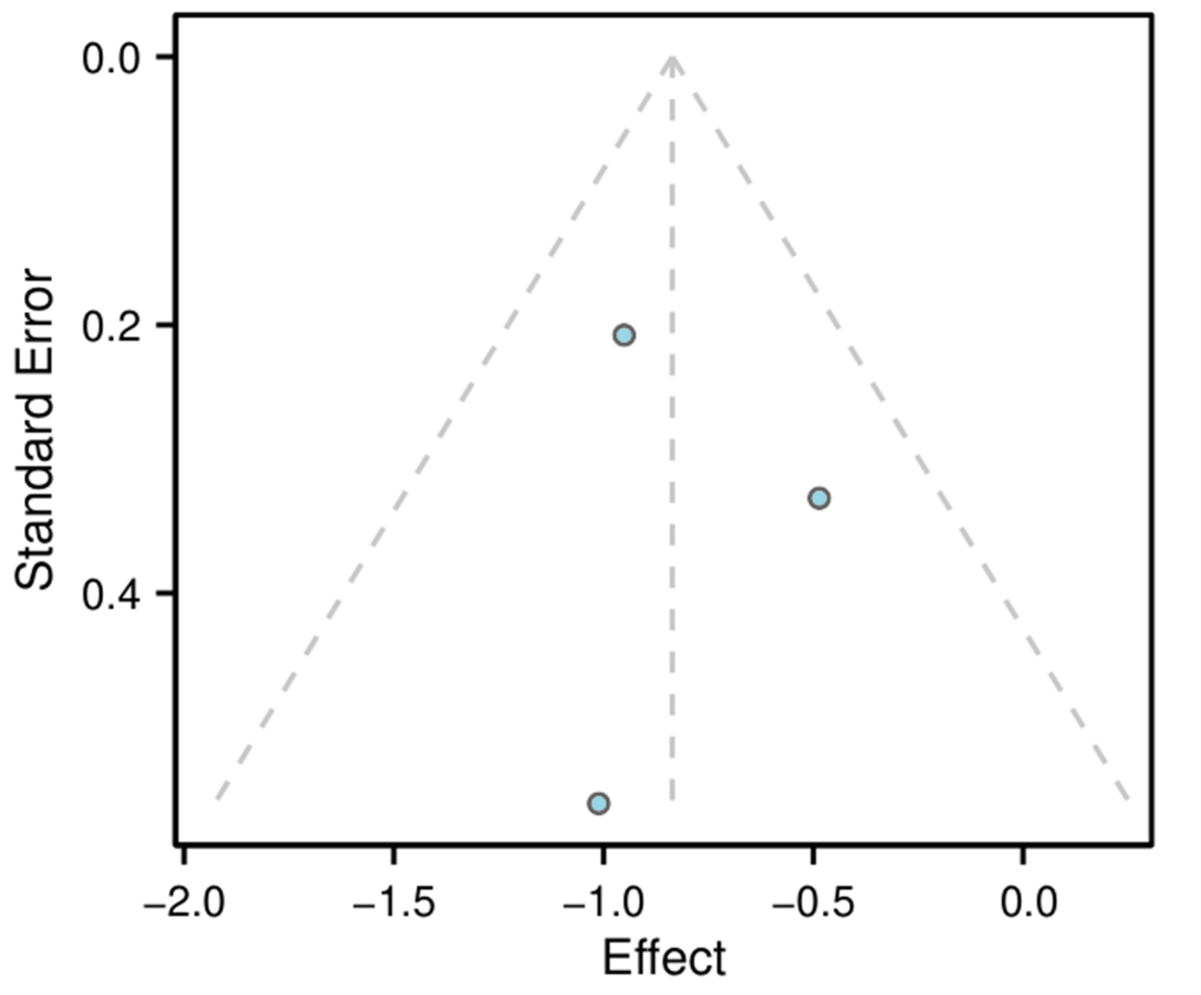
Funnel plot to assess publication bias Funnel plot of effect size (risk ratio) against standard error for the three studies included in the meta-analysis. Asymmetry may suggest publication bias, but the small number of studies makes this difficult to assess definitively

A funnel plot was generated to visually explore the potential for publication bias (Figure 5). Due to the small number of studies (n = 3) included in the meta-analysis, the power to distinguish chance from real asymmetry was limited, and statistical tests for funnel plot asymmetry were not performed.

Sensitivity analysis using the leave-one-out method (Table 2) demonstrated that the direction and significance of the pooled effect estimate were not substantially altered by the removal of any single study, supporting the robustness of the findings.

**Table 2:** Sensitivity analysis (leave-one-out method) Results of the leave-one-out sensitivity analysis for the meta-analysis, showing the stability of the pooled effect estimate when each study is omitted sequentially. Data for this analysis were derived from Ma et al. [10], Zheng et al. [8], and Zhan et al. [7] TE: estimate of the pooled effect; CI lower/upper: confidence interval limits; tau²: estimate of between-study variance; I²: proportion of total variability due to heterogeneity

Study	TE	CI lower	CI upper	Statistic	P	Tau^2^	I^2^
Omitting Ma et al., 2024	0.383517	0.298057	0.49348	-48.3034	0.0131777	0	0
Omitting Zheng et al., 2023	0.536906	0.028778	10.0169	-2.70056	0.22577	0	0
Omitting Zhan et al., 2022	0.454511	0.0271568	7.60694	-3.55597	0.174521	0.0324647	0.300175

Discussion

This systematic review and meta-analysis provide preliminary evidence that esketamine, as an adjunct to propofol, significantly reduces the risk of propofol-related adverse events during gastrointestinal endoscopy. The pooled analysis of three RCTs, involving 524 patients, showed a 57% relative risk reduction (RR: 0.43). The beneficial effect aligns with the known pharmacology of esketamine. Its analgesic and sedative properties likely create a synergistic effect with propofol, reducing the total propofol dose required and thereby mitigating its dose-dependent side effects, particularly respiratory and cardiovascular depression [13]. Furthermore, esketamine's ability to maintain respiratory drive and stabilize blood pressure offers a distinct advantage over other adjuncts like opioids [5].

For the quantitative synthesis, we made a pragmatic decision to pool data only from the three studies that provided complete outcome data suitable for meta-analysis [7-8,10]. The exclusion of the other three studies was necessary: two were study protocols without results [6,11], and one employed a three-arm design [12], which did not align with our planned comparison. Our methodology involved a comprehensive quality assessment of all six identified RCTs, which overall demonstrated a low risk of bias. While a meta-analysis with three studies is limited, it is methodologically acceptable when the studies address a focused question and are sufficiently homogeneous in their design and outcomes [9]. It provides a valuable quantitative summary of the available evidence, guiding clinical practice and future research.

Limitations

Our meta-analysis has several limitations that should be considered when interpreting the findings. First and most notably, the quantitative synthesis included only three RCTs with a relatively small total sample size (n = 524), which limits the statistical power and precision of our pooled estimate. Second, we observed considerable statistical heterogeneity (I² > 50%), which likely stems from clinical variations such as differences in esketamine dosing regimens, patient populations (e.g., obese vs. general), and definitions of adverse events across the included trials. Third, all included studies were conducted in a single country (China), which may affect the generalizability of the results to other populations or healthcare settings. Finally, the inability to assess publication bias reliably due to the small number of studies remains a methodological constraint.

However, despite these limitations, the consistency in the direction of effect across studies and the robustness shown in sensitivity analysis strengthen the credibility of our primary conclusions.

## Conclusions

The findings of this meta-analysis suggest that esketamine may reduce propofol-related adverse events during gastrointestinal endoscopy. The methodological approach of qualitatively synthesizing all eligible studies and quantitatively pooling data from a homogenous subset is justified and provides the most robust possible summary of the current evidence. Future large-scale, multi-center RCTs with standardized outcome measures are needed to strengthen this evidence base and establish optimal clinical protocols.
